# *FLEXR*: automated multi-conformer model building using electron-density map sampling

**DOI:** 10.1107/S2059798323002498

**Published:** 2023-04-18

**Authors:** Timothy R. Stachowski, Marcus Fischer

**Affiliations:** a Department of Chemical Biology and Therapeutics, St Jude Children’s Research Hospital, Memphis, TN 38105, USA; Diamond Light Source, United Kingdom

**Keywords:** crystallographic refinement, model building, multi-conformer, occupancy, conformational flexibility, *FLEXR*

## Abstract

Protein conformational dynamics that may inform biology often lie dormant in high-resolution electron-density maps. While an estimated ∼18% of side chains in high-resolution models contain alternative conformations, these are underrepresented in current PDB models due to difficulties in manually detecting, building and inspecting alternative conformers. To overcome this challenge, we developed an automated multi-conformer modeling program, *FLEXR*. Using *Ringer*-based electron-density sampling, *FLEXR* builds explicit multi-conformer models for refinement. Thereby, it bridges the gap of detecting hidden alternate states in electron-density maps and including them in structural models for refinement, inspection and deposition. Using a series of high-quality crystal structures (0.8–1.85 Å resolution), we show that the multi-conformer models produced by *FLEXR* uncover new insights that are missing in models built either manually or using current tools. Specifically, *FLEXR* models revealed hidden side chains and backbone conformations in ligand-binding sites that may redefine protein–ligand binding mechanisms. Ultimately, the tool facilitates crystallographers with opportunities to include explicit multi-conformer states in their high-resolution crystallographic models. One key advantage is that such models may better reflect interesting higher energy features in electron-density maps that are rarely consulted by the community at large, which can then be productively used for ligand discovery downstream. *FLEXR* is open source and publicly available on GitHub at https://github.com/TheFischerLab/FLEXR.

## Abstract 2

Alternative conformations are underrepresented in current PDB models due to difficulties in manually detecting, building and inspecting multiple conformers. To overcome this shortcoming, an automated multi-conformer modeling program, *FLEXR*, has been developed that uses *Ringer*-based electron-density sampling to explicitly build multi-conformer models for refinement.

## 1. Introduction

Flexibility underscores many aspects of protein function such as ligand binding, catalysis and allostery (Buhrman *et al.*, 2010 ▸; Krojer *et al.*, 2020 ▸; Henzler-Wildman & Kern, 2007 ▸; Eisenmesser *et al.*, 2005 ▸). Rather than a single static structure, proteins exist as an ensemble of states, which repopulate in response to different perturbations and environments (Fraser *et al.*, 2011 ▸; Girard *et al.*, 2022 ▸; Russi *et al.*, 2017 ▸). Developing comprehensive movies of protein motion can communicate a deeper understanding of the relationship between structure and function and reveal new opportunities to design thera­peutics (Carlson, 2002 ▸; Meagher & Carlson, 2004 ▸).

X-ray crystallography is one of the foremost techniques used to develop three-dimensional protein structures with near-atomic spatial resolution. Crystallography is intrinsically an ensemble measurement, where the final electron-density map represents an average across all protein copies in the lattice. Therefore, maps contain information about conformational heterogeneity, which is often transient, sparse and difficult to detect. There has been a recent surge of new and sensitive crystallographic methods that link shifting conformational ensembles to different stimuli such as temperature (Stachowski *et al.*, 2022 ▸; Bradford *et al.*, 2021 ▸), photoactivation (Tenboer *et al.*, 2014 ▸) and ligand binding (Krojer *et al.*, 2020 ▸; Stachowski & Fischer, 2022 ▸; Pearce, Krojer & von Delft, 2017 ▸). For example, electron-density maps developed at elevated temperatures often contain a richer picture of protein dynamics that includes side-chain and backbone repositioning, which is often hidden or frozen out at cryogenic temperatures, at which crystallographic studies are typically performed (Fischer, 2021 ▸). Time-resolved experiments can interrogate femtosecond conformational changes at X-ray free-electron laser facilities (Pandey *et al.*, 2020 ▸) and microsecond dynamics at third-generation synchrotron light sources (Pearson & Mehrabi, 2020 ▸). These experiments are progressing towards ‘molecular movies’ that show complete real-time conformational trajectories (Martin-Garcia *et al.*, 2016 ▸). The recent emergence of high-throughput protein–ligand crystallography can reveal weakly populated ligand-bound states (Pearce, Krojer, Bradley *et al.*, 2017 ▸) and allosteric sites (Krojer *et al.*, 2020 ▸). Together, these new methods are creating large amounts of crystallographic data and require corresponding advances in computational modeling that accurately, thoroughly and quickly describe alternate conformational states that either occur simultaneously or transition from one state to another. While there are several automatic approaches for building single-conformer models (Joosten *et al.*, 2009 ▸; Langer *et al.*, 2008 ▸; Cowtan, 2006 ▸; Terwilliger, 2004 ▸), there are fewer options for building multi-state models.

Conformational heterogeneity in protein crystal structures can be represented by *B* factors, multi-copy and multi-conformer modeling (Riley *et al.*, 2021 ▸; van den Bedem & Fraser, 2015 ▸). Firstly, *B* factors represent the thermal displacement of individual atoms around a mean position isotopically and, at a sufficient ratio of observations to parameters, anisotropically (Merritt, 1999 ▸). Yet, *B* factors also reflect the general uncertainty of the position of each atom and do not describe correlated positional changes of groups of atoms, for example transitions of side-chain rotamers (Lovell *et al.*, 2000 ▸). Secondly, multi-copy modeling produces more than one discrete and complete protein structure to explain a single set of experimental data. This is commonly practiced in cryo-electron microscopy via multi-body refinement (Nakane *et al.*, 2018 ▸), but one tool for crystallographic data is *Phenix-MD* (*phenix.ensemble_refinement*; Burnley *et al.*, 2012 ▸). *Phenix-MD* combines traditional *Phenix* crystallographic refinement with density-restrained molecular-dynamics (MD) simulations that focus on sampling local dynamics. However, MD using crystal structures as simulation starting points can be limited by energy barriers (Ploscariu *et al.*, 2021 ▸), especially with structures solved at cryogenic temperatures (Bradford *et al.*, 2021 ▸). While multi-copy approaches do comprehensively describe conformational heterogeneity, the main drawback is that they often unnecessarily inflate the number of parameters that are needed to describe the data. For instance, many of the atoms in each model that are not flexible will share the same position across copies, so there is no need to describe them more than once. In crystallography, this overfitting is detrimental and will overextend the data-to-parameter ratio and increase *R*_gap_ (Ginn, 2021 ▸). Lastly, multi-conformer modeling aims to explain both areas of conformational heterogeneity and homogeneity in a single parsimonious model (Keedy, 2019 ▸). A popular implementation of this is *qFit*, which has been developed through several iterations (Keedy *et al.*, 2015 ▸; van den Bedem *et al.*, 2009 ▸) and expanded to interrogate protein, ligand (van Zundert *et al.*, 2018 ▸) and cryo-EM data (Riley *et al.*, 2021 ▸). *qFit* works by computing an occupancy-weighted set of both main-chain and side-chain conformations that together best describe the electron density. *qFit* initially creates many conformations using mixed-integer quadratic programming and iteratively whittles the possibilities down to a handful of conformers using a convex optimization algorithm (Keedy *et al.*, 2015 ▸).

Another tool is *Ringer*, which falls into its own class of ‘conformation-detection’ programs (Lang *et al.*, 2010 ▸). *Ringer* measures the electron density around side-chain dihedral angles, where peaks in density correspond to side-chain conformations. *Ringer* was used to systematically interrogate a subset of the PDB with resolution better than 1.5 Å and it was found that a surprisingly high number (∼18%) of side chains have alternative conformations, many of which are not accounted for in the deposited models (Lang *et al.*, 2010 ▸). *Ringer* detects weakly populated rotamers in electron density down to 0.3σ and can interrogate conformational changes from temperature (Stachowski *et al.*, 2022 ▸) and ligand binding (Bradford *et al.*, 2021 ▸) and validate cryo-EM models (Barad *et al.*, 2015 ▸). While *Ringer* typically requires complete manual inspection of per-residue ‘Ringer plots’, we have recently automated peak detection to highlight regions of interest and reveal ‘conformational barcodes’ (Stachowski *et al.*, 2022 ▸). Yet, for all its usefulness, explicitly including information from *Ringer* into protein models still requires tedious manual model building.

To bridge this gap, we combined our previous *Ringer* peak-finding tools with the model-building functions of *Coot* (Emsley & Cowtan, 2004 ▸) into a pipeline: *FLEXR*. *FLEXR* automatically detects alternative side-chain conformations in *Ringer* measurements and builds them into protein models. To assess its utility, we compared *R* factors, geometry metrics and properties of side chains in *FLEXR* models against deposited and *qFit*-derived models using a test set of high-quality X-ray structures. We also inspected ligand-binding sites and found examples where *FLEXR* detected and built minor side-chain conformations that are missing in models produced by other methods. Although not inherently detected by *Ringer*, combining models from *FLEXR* with traditional refinement exposed examples of backbone heterogeneity. Ultimately, *FLEXR* simplifies initial and systematic examinations of protein flexibility in crystal structures and, more importantly, enables this information to be automatically incorporated in deposited models for the community at large. To facilitate this, we have made *FLEXR* freely available at https://github.com/TheFischerLab/FLEXR.

## 2. Materials and methods

### 2.1. *FLEXR* algorithm

#### 2.1.1. Overview

**Figure 1:**
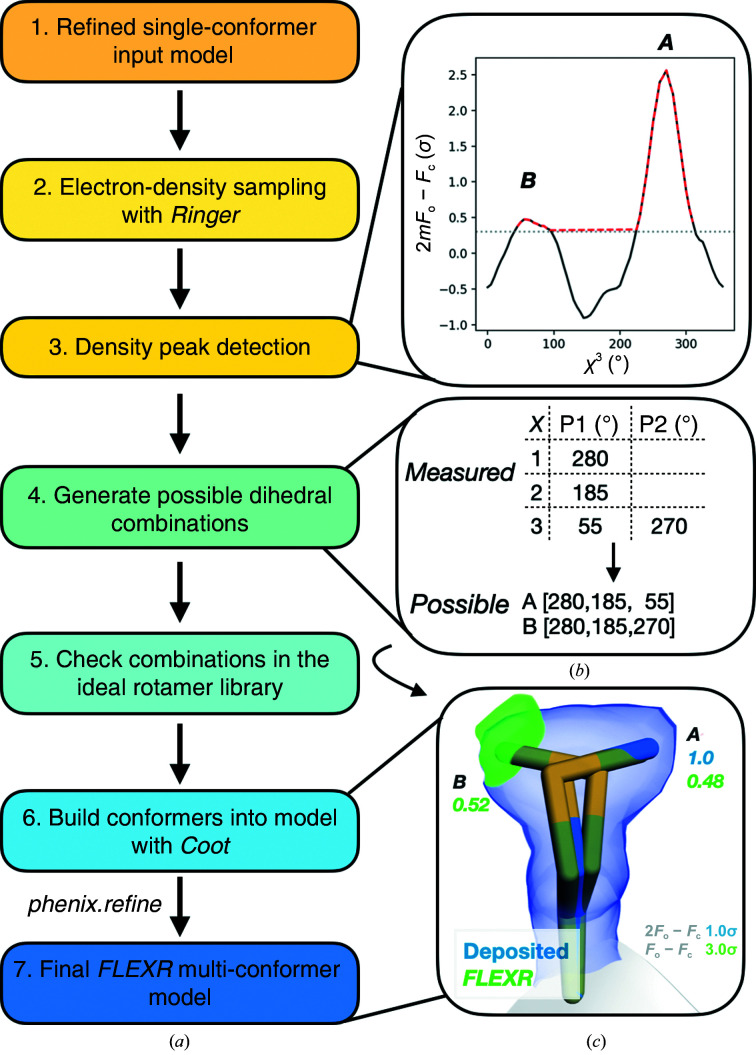
*FLEXR* workflow. *FLEXR* builds multi-conformer models based on electron-density sampling around side-chain dihedrals (χ) performed by *Ringer*. (*a*) *FLEXR* detects peaks in the electron-density measurements above a user-defined σ threshold (default 0.3σ). Peak angles from each dihedral are assembled into possible rotamers and checked against the ideal rotamer library (Lovell *et al.*, 2000 ▸). Valid alternate conformers are automatically built using *Coot*. Refining the multi-conformer models estimates occupancies and improves the geometry. (*b*) An example of the workflow is shown for Met313 in prostaglandin reductase 3 (MGC45594), which is modeled in a single conformation in the A chain of the 1.45 Å resolution structure (PDB entry 2c0c). Top: two peaks (stars) are detected in electron density sampled around χ^3^ using data > 0.3σ (red dashed line). Bottom: combining the detected peaks across the three dihedrals yields two possible rotamers. (*c*) After automated building with *Coot*, a visual inspection shows that the two rotamers (green sticks) satisfy the deposited electron density. A final refinement with *Phenix* shows that the two rotamers have roughly equal occupancies. The deposited 2*mF*_o_ − *DF*_c_ map (blue) is contoured at 1σ and the *mF*_o_ − *DF*_c_ difference density map (green) is contoured at 3σ.

*FLEXR* automatically finds and builds alternative side-chain conformations into models based on *Ringer* electron-density measurements (Fig. 1 ▸). *FLEXR* is a command-line tool and is written in Python (version 3.9) using the *Pandas*, *SciPy* and *NumPy* packages. Automatic model building is performed using *Coot* (version 1.0.05; Emsley & Cowtan, 2004 ▸; Casañal *et al.*, 2020 ▸; Emsley *et al.*, 2010 ▸). *FLEXR* was tested on MacOS Monterey with Apple M1 and Intel processors. Instructions on how to use *FLEXR* can be found in the supporting information and at https://github.com/TheFischerLab/FLEXR.

#### 2.1.2. *FLEXR* Part 1: electron-density peak detection

*FLEXR* relies on the original *Ringer* algorithm for electron-density sampling around each dihedral of each residue in the input model (Lang *et al.*, 2010 ▸). *Ringer* is available through the *mmtbx* module in the *cctbx* library (Grosse-Kunstleve *et al.*, 2002 ▸). *FLEXR* uses the peak-detection algorithm that we have recently implemented (Stachowski *et al.*, 2022 ▸) to find heightened levels of density that correspond to potential alterative side-chain positions. The angle where each density peak occurs is recorded.

#### 2.1.3. *FLEXR* Part 2: assembling peaks into side-chain rotamers

The angle for each detected peak from Part 1 is assembled across all dihedrals for a side chain into all possible combinations. Each combination is tested using the ideal rotamer library (Lovell *et al.*, 2000 ▸) to determine whether the measured dihedral angles are close to ideal rotameric angles based on a user-defined threshold (see below). Combinations that have tolerable geometry are assigned a ‘rotamer name’ from the matching entry, for instance ‘p’, ‘t’ or ‘m’ for the three ideal threonine rotamers, and will be incorporated based on this name into the model using *Coot* in Part 3. In the case where a measured rotamer matches multiple entries in the rotamer library, the rotamer that is most frequent in the PDB is selected for building. One caveat of this approach is that determining rotamers from density peaks alone is inherently ambiguous. For example, a residue with two dihedrals and two peaks at each dihedral creates up to four possible rotamers, but no less than two. This situation is exacerbated at moderate resolutions and especially with flexible residues such as lysine and arginine that have four dihedrals and often do not have well resolved electron density at higher dihedrals. *FLEXR* is designed to systematically build all possible rotamers (with reasonable geometry). Therefore, we strongly recommend that users refine the final model and manually inspect alternative conformers.

#### 2.1.4. *FLEXR* Part 3: multi-conformer model building

The alternative side-chain conformations assembled in Part 2 are automatically built into models using *Coot*. *FLEXR* automatically iterates through options that are available in the familiar GUI windows used for manual model building. Conformers are added according to the ‘rotamer name’ assigned in Part 2. Users have the option to build conformers beginning at the C^α^ atom or with the entire residue including backbone atoms.

#### 2.1.5. *FLEXR* Part 4: refinement

*FLEXR* multi-conformer models can be refined using any macromolecular refinement program that the user desires. Here, we used *Phenix* to improve geometries and estimate occupancies and used ten macrocycles with default settings and water picking followed by three refinement cycles that optimize the *B* factor and coordinate weighting.

### 2.2. Evaluating *FLEXR*

To test *FLEXR*, we used a set of 15 models with resolutions ranging from 0.80 to 1.85 Å that was first curated by Keedy and coworkers to evaluate *qFit* versions 2.0 (Keedy *et al.*, 2015 ▸) and 3.0 (Riley *et al.*, 2021 ▸). We chose this established benchmarking set as it contains high-quality data sets with missing alternative side-chain conformations and includes biomedical and ligand-bound targets. Coordinates and structure factors were obtained from the Protein Data Bank (Burley *et al.*, 2022 ▸). Alternate conformations in the deposited models were discarded prior to multi-conformer modeling. The deposited models were compared using the original deposited models (‘deposited’) and the deposited models after refinement using the same *Phenix* protocol as the *FLEXR* models (‘dep-refined’). *FLEXR* was performed with a peak-detection threshold of σ ≥ 0.35 and a tolerance for matching ideal rotameric geometries of ≤30°. The values of both parameters are close to those suggested by Lang *et al.* (2010 ▸) to maximize sensitivity while avoiding false positives. *FLEXR* models were refined as described in Section 2.1.5. Alternate side-chain conformations were built using completely new residues (as opposed to branching at the C^α^ atom). *qFit* (version 3.0) was performed with backbone modeling (‘*qFit’*) and without (using the --no-backbone option; ‘*qFit*-no-bb’), using default settings otherwise. *qFit* models were refined using their packaged refinement script (qfit_final_refine_xray.sh) using default settings. *Phenix* (version 1.20) was used throughout for refinement and *R*-value calculations (Liebschner *et al.*, 2019 ▸). Model-quality metrics were calculated with *MolProbity* (Williams *et al.*, 2018 ▸). *Phenix* tools were used to calculate real-space correlation coefficients (RSCCs; *phenix.real_space_correlation*) and rotamer geometries (*phenix.rotalyze*). Normalized *B*-factor values (*B*_norm_) were calculated according to *B*_norm_ = (*B* − *B*_μ_)/*B*_σ_, where *B*_μ_ is the mean and *B*_σ_ is the standard deviation. *B*_norm_ values were calculated using all protein atoms. Other alternate side-chain characteristics were calculated using custom Python scripts. Images were rendered using *PyMOL* (Schrödinger).

## 3. Results

### 3.1. Validation of rotamers in *FLEXR* models

**Figure 2:**
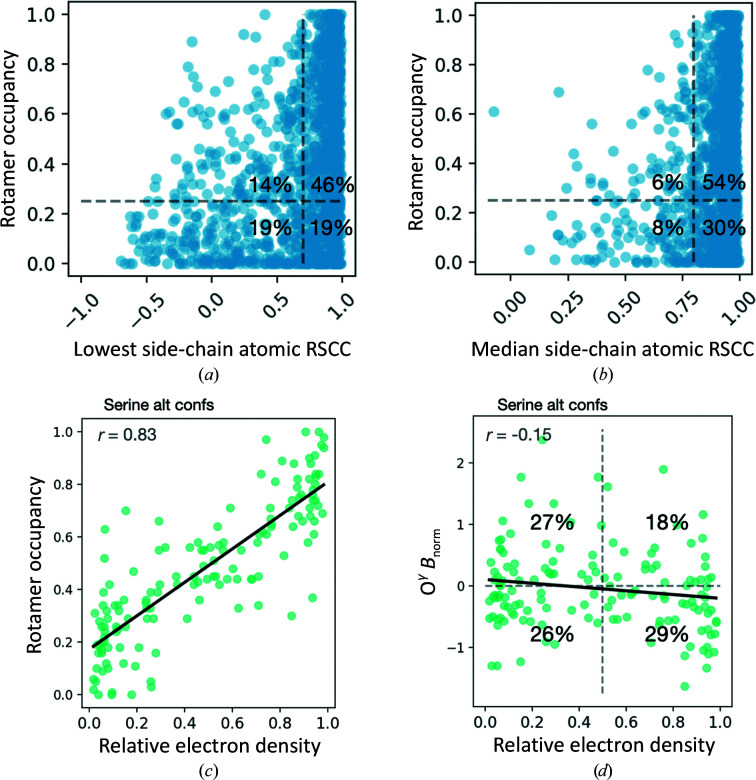
Map–model fit properties validate the newly built rotamers. (*a*) Comparison of side-chain rotamer occupancy and the lowest atomic real-space correlation coefficient (RSCC; considering non-H atoms), where the vertical gray dotted line is atom RSCC = 0.7 (which represents the cutoff suggested by *MolProbity*) and the horizontal gray dotted line is occupancy = 0.25. (*b*) Comparison of side-chain rotamer occupancy with the median side-chain atom RSCC, where the vertical gray dotted line is RSCC = 0.8 (confident modeling; Burley *et al.*, 2022 ▸) and the horizontal gray dotted line is occupancy = 0.25. (*c*, *d*) The relationship between (*c*) rotamer occupancy or (*d*) O^γ^ *B*_norm_ and the relative electron-density peak area of serine rotamers. To calculate the relative peak area, peaks from *Ringer* plots were integrated and normalized across peaks of the same residue. Data were fitted to a linear regression model, where *r* is the Pearson correlation coefficient.

To evaluate the performance of *FLEXR* we used a test set of 15 high-resolution X-ray structures (Keedy *et al.*, 2015 ▸). Running on a single processor, detecting and building side-chain conformers with *FLEXR* took ∼2 min for most structures. Notably, this step only trivially increased the total average time of 107 min spent to refine these structures with *Phenix* (Supplementary Fig. S1). To validate the placement of rotamers in *FLEXR* models, we monitored the real-space correlation coefficient (RSCC), normalized *B* factors (*B*_norm_) and occupancies. Some 65% of rotamers have all side-chain atoms with RSCC > 0.70 (the suggested cutoff for modeling by *MolProbity*) and 19% of these rotamers have low occupancies (occupancy < 0.25; Fig. 2 ▸ *a*). Meanwhile, 85% of rotamers had a median side-chain atom RSCC > 0.8, 30% of which have low occupancies (Fig. 2 ▸ *b*). Similarly, less than 14% of rotamers exhibited high (>1) *B*_norm_ values (Supplementary Fig. S2). Interestingly, some of the rotamers with the highest *B*_norm_ values had reasonable occupancy and RSCC values.

Considering that the proportions of alternative conformations are often inferred from *Ringer* peaks without being directly estimated from refinement, we next sought to test the relationship of occupancies to *Ringer* electron-density measurements. Since serines have a single, unbranched dihedral angle they present the most straightforward test case. Comparing refined occupancies with integrated electron-density peak areas relative to conformers of the same residue in *FLEXR* models showed a strong positive relationship with a Pearson correlation coefficient (*r*) of 0.83 (Fig. 2 ▸ *c*). Unsurprisingly, this relationship was weakest where electron-density peaks were also weak. For example, electron-density peaks with relative areas varying between 0.01 and 0.10 generally varied in refined occupancy between 0.00 and 0.40 and sometimes exceeded 0.60. Additionally, relative electron density does not appear to be a strong predictor of refined *B*_norm_ (*r* = −0.15; Fig. 2 ▸ *d*). For example, 26% of rotamers identified from weak *Ringer* peaks refined with low *B*_norm_ values, whereas 18% of rotamers identified from strong peaks had large *B*_norm_ values.

**Figure 3:**
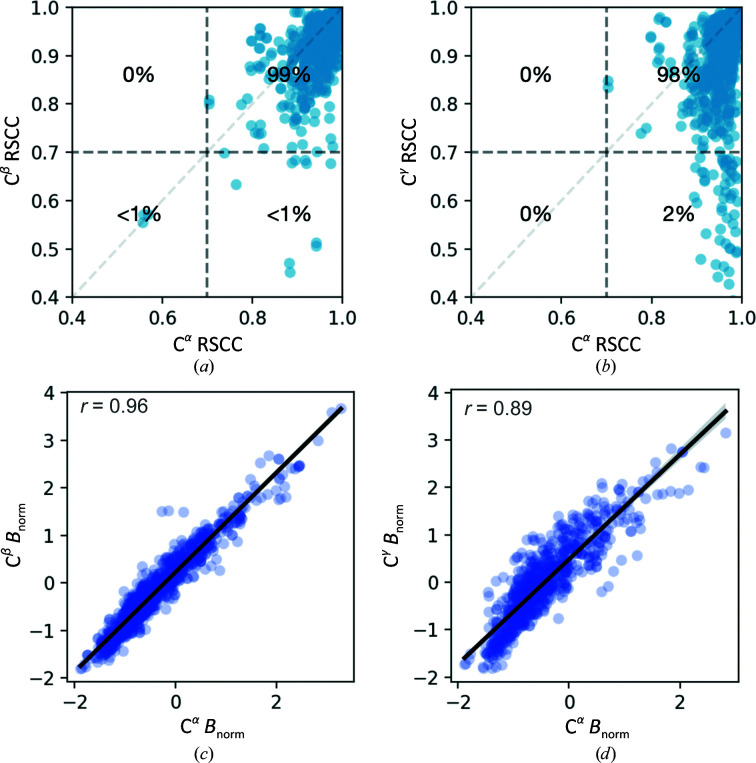
Atomic properties validate newly built rotamers. (*a*, *b*) The relationship between C^α^ and (*a*) C^β^ or (*b*) C^γ^ atoms of the respective rotamer (dark gray dotted lines, RSCC = 0.7). (*c*, *d*) The relationship between *B*_norm_ of C^α^ and (*c*) C^β^ or (*d*) C^γ^ atoms. The solid black line represents a fit of the data to a linear regression model, where *r* is the Pearson correlation coefficient.

Flexible residues such as arginine and lysine and aromatic residues are the most poorly modeled residues, while the sulfur-containing residues (cysteine and methionine) and small residues (such as serine and threonine) were modeled best (Supplementary Fig. S3). Yet, relating RSCC (Figs. 3 ▸ *a* and 3 ▸ *b*) and *B*_norm_ (Figs. 3 ▸ *c* and 3 ▸ *d*) of C^α^ atoms to those of the more distant side-chain C atoms C^β^ and C^γ^ showed strong correlations throughout. Only 1–2% of side chains contained C^α^ and C^β^ or C^γ^ with RSCC values both below 0.7. This suggests that while there might not be density to support the modeling of entire side chains there is sufficient electron density to identify flexibility at most sites. Inspecting the two side chains with the most egregious C^β^ RSCC values showed that these side chains still have convincing density for the placement of an alternative conformation (Supplementary Fig. S4).

### 3.2. Improved multi-conformer model quality with *FLEXR*

**Figure 4:**
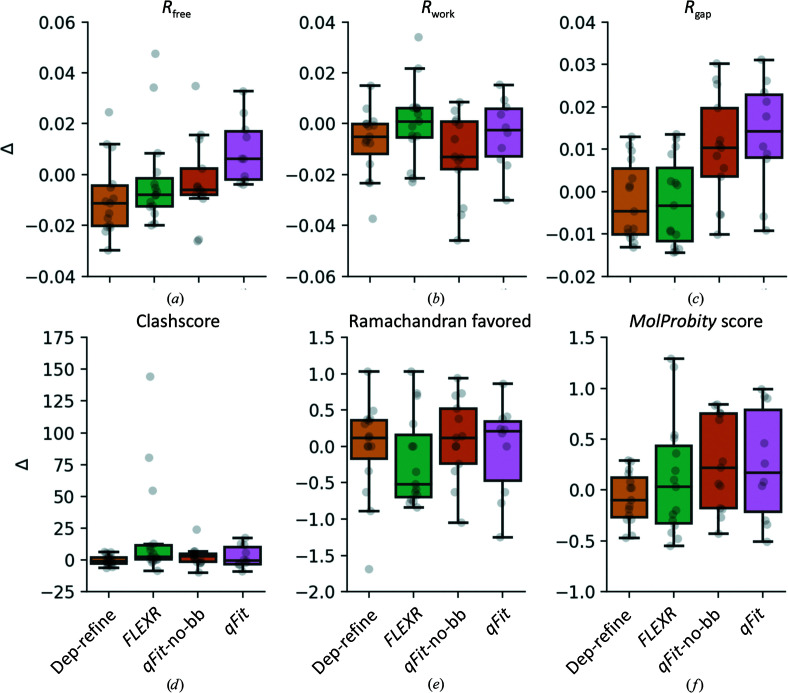
Multi-conformer modeling with *FLEXR* results in similar or better model-quality metrics than other methods. A test set of 15 high-resolution models (Keedy *et al.*, 2015 ▸) was used to compare changes in each metric relative to the original deposited models: (*a*) *R*_free_, (*b*) *R*_work_, (*c*) *R*_gap_, (*d*) clashscore, (*e*) percentage of side chains with favorable Ramachandran geometry and (*f*) *MolProbity* score. We compared five different modeling approaches: deposited models (used as a reference, not shown), re-refined deposited models (light orange), *FLEXR* (green), *qFit* with no backbone sampling (*qFit*-no-bb; dark orange) and *qFit* with backbone sampling (pink). The values shown (Δ) were calculated by taking the difference between the property of the target model and that of the deposited model. Raw values for individual models are given in Supplementary Figs. S5–S8 and S10-S11.

To assess model quality, we compared five different variations of model building using the same 15 data sets: (i) deposited models, (ii) deposited models re-refined with the *Phenix* protocol outlined in Section 2 (‘dep-refined’), (iii) *FLEXR*, (iv) *qFit* without backbone sampling (‘*qFit*-no-bb’) and (v) *qFit*. *B*-factor refinement was treated identically for each model across protocols except in one case (Supplementary Table S1). *qFit* did not converge into a parsimonious model in five of 15 cases (Supplementary Fig. S5); *FLEXR* generated models for all 15 cases. With neither *qFit* approach converging for one case, PDB entry 1w0n, this left 14 cases with at least one *qFit* model. Firstly, we compared how the *R* values of the various modeling approaches deviate from the original deposited models (Fig. 4 ▸). Re-refining the deposited structures produced models with the lowest *R*_free_ in nine out of 14 cases. *qFit* without backbone sampling produced models with a lower *R*_free_ than when including backbone sampling in seven out of ten cases (Supplementary Fig. S5). Relative to either *qFit* approach, *FLEXR* models had a lower *R*_free_ in nine out of 14 cases and for PDB entry 2c6z *FLEXR* produced the model with the lowest *R*_free_ overall (Fig. 4 ▸ *a* and Supplementary Fig. S5). The median *FLEXR* *R*_free_ was 0.002 less than that for the deposited models, 0.01 greater than that for dep-refined models, equal to that for *qFit*-no-bb models and 0.004 lower than that for *qFit* models. As expected, both *qFit* approaches typically produced models with lower *R*_work_ than the deposited and *FLEXR* models (Fig. 4 ▸ *b* and Supplementary Fig. S6), and the *qFit*-no-bb models generally had the lowest *R*_work_ overall. *FLEXR* models have the smallest *R*_gap_ in five out of 15 cases and in 12 out of 14 comparisons to either *qFit* models (Fig. 4 ▸ *c* and Supplementary Fig. S7). The clashscores for *FLEXR* models were close (<5 points) to the deposited models in ten out of 15 cases and lower than the respective *qFit* model in four out of 14 cases (Fig. 4 ▸ *d* and Supplementary Fig. S8). Yet, three *FLEXR* models had egregiously high clashscores, with one even exceeding a value of 100, whereas deposited models all had scores of less than 12. Inspecting some of these cases showed that weak alternative conformations were built in the interior of the protein in impossible positions despite having *Ringer* peaks at rotameric angles (Supplementary Fig. S9). Notably, the *FLEXR* models with high clashscores were at higher resolutions. *FLEXR* models also generally had poorer Ramachandran statistics, which were the worst values overall in five out of 15 cases (Fig. 4 ▸ *e* and Supplementary Fig. S10). Despite this, in terms of *MolProbity* scores, which compress many validation metrics, including clashscore, into a single score, *FLEXR* models scored well overall. Lower scores correspond to better models and *FLEXR* models had a median score of 1.66, which falls between those for dep-refined (1.58) and *qFit*-no-bb models (1.74). *qFit* models have the highest (worst) scores overall, with a median of 1.86 (Fig. 4 ▸ *f* and Supplementary Fig. S11). Taking the final *FLEXR* models and re-refining them with the *qFit* refinement protocol, which contains an occupancy-based rotamer-culling step, removed many of these problematic side chains but notably produced models with worse *R*_free_ values (Supplementary Figs. S12 and S13). Together, these results show that multi-conformer models produced by *FLEXR* typically have similar or better model quality metrics compared with models produced by the two *qFit* approaches that we tested.

### 3.3. *FLEXR* produces models with diverse side-chain populations

**Figure 5:**
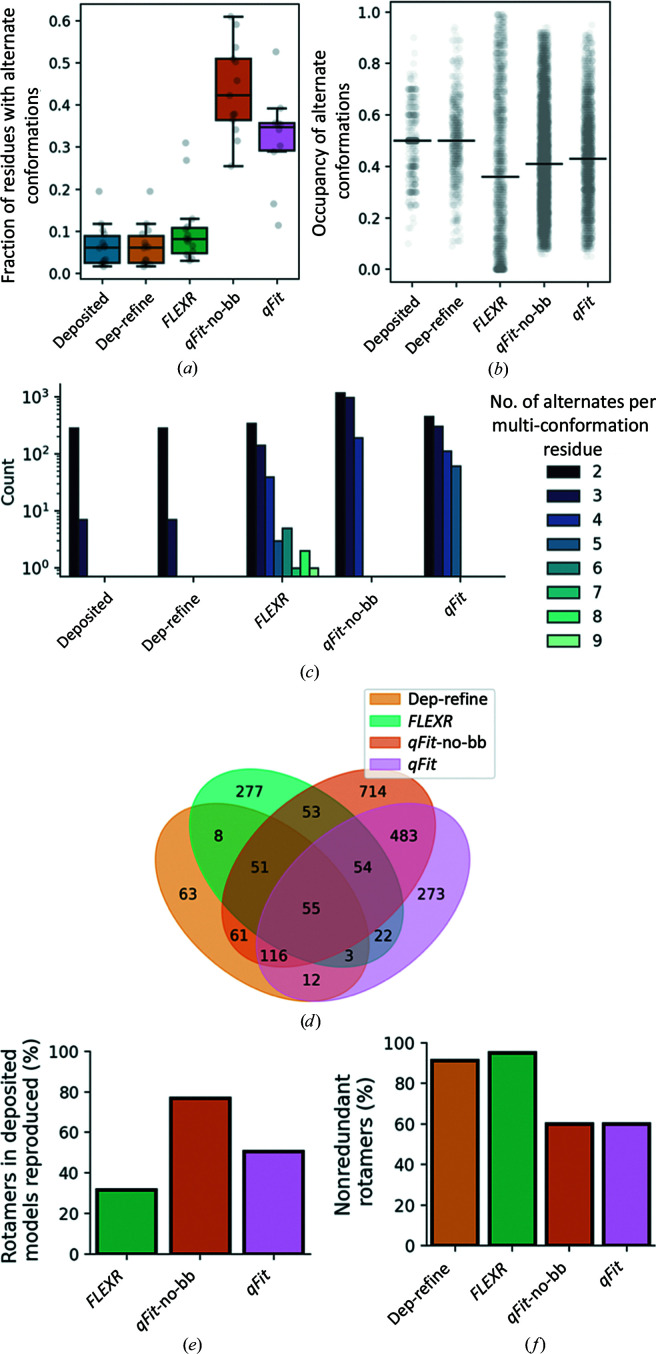
*FLEXR* produces different multi-conformer models. (*a*) The fraction of residues with alternative conformations in deposited models (light blue), re-refined deposited models (light orange), *FLEXR* (green), *qFit* with no backbone modeling (dark orange) and *qFit* (pink). (*b*) The distribution of occupancies of alternative side-chain conformations; bars indicate medians. (*c*) Distribution of the number of alternative conformations for each residue with more than one conformation (*i.e.* multi-conformer residues), on a log scale. Values for individual models are given in Supplementary Figs. S14–S16. (*d*) Venn diagram of rotamers shared between the structures (*n* = 9) across modeling methods. (*e*) The percentage of rotamers produced by *qFit* and *FLEXR* that match rotamers present in the deposited models. (*f*) The percentage of non-identical rotamers.

To evaluate the model details, we next compared how side chains were modeled across methods. In the original *Ringer* paper, the authors estimated that ∼18% of side chains in models with resolutions <1.5 Å had alternative conformations (Lang *et al.*, 2010 ▸). As expected, the deposited models typically have ∼5% of side chains with modeled alternative conformations (Fig. 5 ▸ *a* and Supplementary Fig. S14). While *FLEXR* models have more alternate conformers, by <10% on average, these are still much fewer than the anticipated 18%, but this could be caused by the lower resolution (up to 1.85 Å) of the test set that we used. However, *qFit*-no-bb models detected a median of >40% alternate conformers, while *qFit* had a median of ∼35%, both of which are much higher than the expected frequency. Looking at the number of conformations per residue with multiple conformations shows that most deposited models typically contain two alternative conformers (for example A and B) and in these cases usually have even occupancies, suggesting that occupancies were not refined prior to deposition (Figs. 5 ▸ *b* and 5 ▸ *c* and Supplementary Figs. S15 and S16), which may explain the improvement of *R*_free_ in deposited models using our simple refinement protocol (Fig. 4 ▸ *a*). This is also supported by the more heterogeneous distribution of occupancies after refinement (Fig. 5 ▸ *b*). Median occupancies in both *qFit* approaches are also generally lower than in the deposited models (Fig. 5 ▸ *b*) due to the larger number of built conformers compared with the other methods (Fig. 5 ▸ *a*). While side chains in *FLEXR* models have the lowest median occupancy, this is deflated by the 8% of conformers with occupancies of zero (Supplementary Fig. S17). We recommend removing zero-occupancy conformers prior to the final refinement as described in the supporting information; including them can lead to issues with occupancy refinement, bulk-solvent masking, map calculation and interpretation. Almost half of all zero-occupancy conformers were found in PDB entry 1x9i (Supplementary Fig. S17). Counting the number of alternate conformations per multi-conformer residue further reveals the differences between manual model building, *FLEXR* and *qFit* approaches (Fig. 5 ▸ *d*). Again, most manually built models generally use only two alternative conformations to explain side-chain flexibility. *qFit* models contain a larger proportion of side chains with 3–4 conformations, and surprisingly *qFit* with backbone sampling allows some side chains to be modeled with up to five conformers. *FLEXR* models have the most heterogenous side-chain modeling as most side chains with alternative states contain 2–4 conformers and, rarely, up to nine (Supplementary Figs. S15 and S16). Comparing the commonality of rotamers revealed that surprisingly few rotamers were found in all methods and each method produced many rotamers that were unique (Fig. 5 ▸ *d*). Specifically, *FLEXR* reproduced 32% of rotamers present in deposited models and *qFit* and *qFit*-no-bb reproduced 50% and 77%, respectively (Fig. 5 ▸ *e*). However, *FLEXR* produced the highest percentage of non-identical rotamers at 91% compared with ∼60% for both *qFit* approaches (Fig. 5 ▸ *f*).

### 3.4. *FLEXR* reveals new side-chain conformations at protein–ligand interfaces

**Figure 6:**
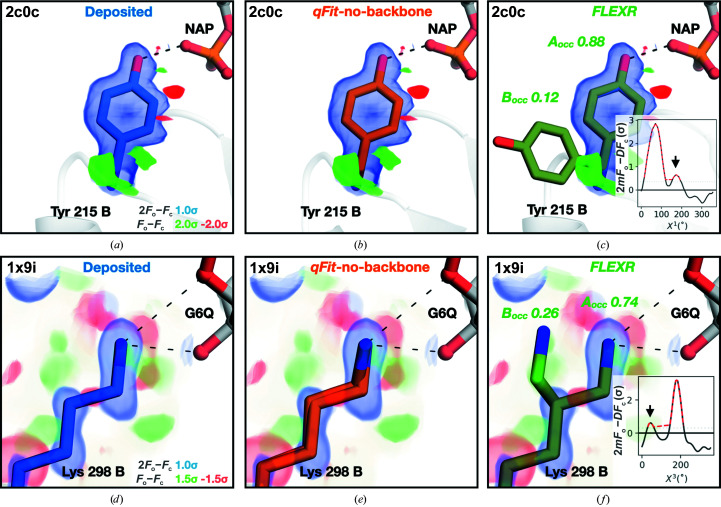
*FLEXR* models reveal side-chain repositioning in ligand-binding sites that is missing in other models. (*a*) Tyr215 in the B chain of the 1.45 Å resolution prostaglandin reductase 3 (MGC45594) structure bound to NAP (PDB entry 2c0c). The *qFit* (*b*) and deposited (*a*) models both share the A conformation. (*c*) *FLEXR* detects and models a weak conformation (arrow in the *Ringer* plot) of Tyr215 oriented away from and no longer hydrogen bonding to the ligand. This feature is not present in either the deposited or *qFit* models. (*d*) Lys298 in the B chain of the 1.16 Å resoluton *Pyrobaculum aerophilum* phosphoglucose isomerase structure bound to glucose 6-phosphate (G6Q; PDB entry 1x9i). (*e*) *qFit*-no-bb produces two near-identical rotamers. (*f*) *FLEXR* detects a minor side-chain conformation (arrow in the inset *Ringer* plot) which is oriented away from and no longer interacts with the ligand. The deposited 2*mF*_o_ − *DF*_c_ maps are contoured at 1.0σ and the *mF*_o_ − *DF*_c_ maps are contoured at −2.0σ/2.0σ (*a*) or −1.5/1.5σ (*c*, *d*, *e*). Dotted lines represent predicted hydrogen bonds.

To explore the sensitivity of each method, we manually investigated flexibility in ligand-binding sites. We identified two instances where *FLEXR* finds hidden side-chain conformations that are missing in the deposited and *qFit* models and would likely have an impact on how ligand binding is interpreted. The first example is Tyr215 in the B chain of the 1.45 Å resolution structure of prostaglandin reductase 3 (MGC45594) bound to NAP (PDB entry 2c0c; Fig. 6 ▸ *a*). In the deposited and *qFit* models (Fig. 6 ▸ *b*) there is a single conformation of Tyr215 (occupancy of 1.0) which hydrogen bonds to NAP. Yet, inspection of the electron density around χ^1^ with *Ringer* shows a second minor conformation (Fig. 6 ▸ *c*). This is automatically detected with *FLEXR* and built into the model, which reveals that in this minor conformation the tyrosine is positioned away from and no longer interacts with the ligand 12% of the time. The second example is Lys298 in the B chain of the 1.16 Å resolution *Pyrobaculum aerophilum* phosphoglucose isomerase structure complexed with glucose 6-phosphate (PDB entry 1x9i). Despite clear electron density, the deposited model contains a single conformation of the lysine (occupancy of 1.0) that forms two hydrogen bonds to the ligand (Fig. 6 ▸ *d*). *qFit* built two, apparently redundant, conformations of the lysine (occupancies of 0.69 and 0.31; Fig. 6 ▸ *e*). *FLEXR* detects a second peak in the χ^3^ *Ringer* plot and builds the conformer accordingly (occupancies of 0.74 and 0.26; Fig. 6 ▸ *f*). The weak conformer is positioned away from the ligand and no longer interacts with the ligand 26% of the time based on refined occupancies. These examples show that *FLEXR* can find and build hidden side-chain conformations and enable a more rigorous investigation of protein–ligand interactions.

### 3.5. *FLEXR* can reveal backbone conformational heterogeneity

**Figure 7:**
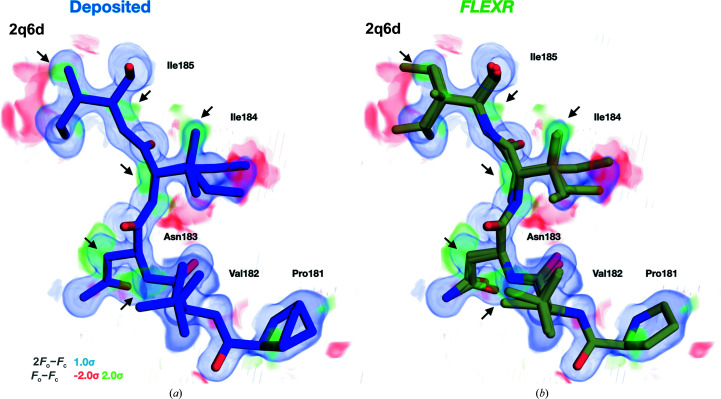
*FLEXR* models reveal backbone heterogeneity. *FLEXR* includes the option to model alternate side-chain conformations starting at the C^α^ atom or create an entirely new residue. Model building using the latter approach and refining the models can reveal alternative backbone orientations. One example of this is the ligand-binding site loop containing Pro181–Ile185 in the B chain of the 1.28 Å resolution HIV-1 protease I50V mutant structure (PDB entry 2qd6). (*a*) The deposited structure has several alternate side-chain conformers but no backbone heterogeneity. (*b*) *FLEXR* recapitulates these alternative side-chain conformations, finds additional ones and reveals backbone conformational heterogeneity. The deposited 2*mF*_o_ − *DF*_c_ map is contoured at 1σ (blue) and the *mF*_o_ − *DF*_c_ map is contoured at 2.0σ (green) and −2.0σ (red). Arrows indicate areas of conformational heterogeneity.

*Ringer* measurements are limited to side-chain dihedrals; they do not provide any information on glycine and alanine residues or backbone conformations. To overcome the limitation of only modeling side-chain heterogeneity, we have given users the option to build in backbone atoms when adding alternative conformations as opposed to adding conformers beginning from the C^α^ atom. Aside from improving geometries, this can reveal backbone conformational heterogeneity when combined with conventional crystallographic refinement of the *FLEXR* multi-conformer model. This feature is most powerful in cases where adjacent residues have alternative side-chain conformations. One example of this is a ligand-binding site loop formed by Pro181–Ile185 in the B chain of the 1.28 Å resolution structure of HIV-1 protease mutant bound to the inhibitor GRL-98065 (PDB entry 2qd6; Fig. 7 ▸). The deposited model contains a single backbone conformation, although most side chains contain multiple conformers. *qFit* did not converge to a multi-conformer model for this data set. The *FLEXR* model recapitulates most of the conformers contained in the original model and adds some additional weakly populated ones. By building in backbone atoms, *FLEXR* enables conventional refinement of backbone atoms to reveal conformational heterogeneity.

## 4. Discussion

Emerging crystallographic methods attempt to link conformational ensembles to function, especially as conformations repopulate in response to changing environments. These structural transitions are often subtle and require sensitive and accurate computational modeling tools. *Ringer* is a popular approach that successfully identifies conformational changes in a variety of contexts. Yet, the insights are not readily incorporable into protein models. This is problematic for two reasons. Firstly, it means that flexibility is underrepresented in deposited models and, since PDB users are often unaware of the specifics of the original study, such structural nuances are lost. Secondly, any flexibility incorporated into the deposited models can be biased by the original scientific question (Wankowicz *et al.*, 2022 ▸). To enable modeling of hidden, low-occupancy conformations in electron-density maps measured by *Ringer*, which is not currently possible, we combined tools that detect conformations in *Ringer* plots and *Coot* model-building functions into an automated multi-conformer model-building tool, *FLEXR*. Using a high-quality test set of 15 models, we validated the placement of *FLEXR* rotamers and compared the results of *FLEXR* with the original, deposited models and *qFit* models. The method is quick, and the longest step remains conventional crystallographic refinement. Three main strengths of *FLEXR* emerge from this work. Firstly, despite its simplicity, *FLEXR* generally performed similarly or better than other methods based on model validation and quality metrics, while avoiding overfitting. Secondly, the side-chain conformations in the models produced by *FLEXR* were more heterogeneous than other models. Thirdly, *FLEXR* captured conformations in ligand-binding sites that remained undetected using other methods.

Modeling conformational ensembles requires extensive parameterization and is generally prohibitive even at high resolutions (Levin *et al.*, 2007 ▸; Burnley *et al.*, 2012 ▸). As such, while modeling additional states can better explain the electron density, *R*_free_ metrics usually do not improve significantly (Keedy *et al.*, 2015 ▸). Yet, nine out of 15 *FLEXR* models exhibited a lower *R*_free_ than their *qFit* counterpart. What could be perceived as a limitation of *Ringer*, *i.e.* its restriction to sampling side-chain dihedrals, is perhaps one of the strengths of the approach. *FLEXR* focuses only on local flexibility without making unnecessary alterations to other areas of the structure and, in the process, avoids overfitting. This is supported by the drastically smaller *R*_gap_ and the higher percentage of nonredundant rotamers in *FLEXR* models compared with *qFit* models. Due to the rigidifying effect of crystal packing (Halle, 2002 ▸) or ligand binding (Wankowicz *et al.*, 2022 ▸), this restriction to local flexibility might be inconsequential for many proteins or at least might be a reasonable starting point for interrogating flexibility. For example, in large surveys of holo–apo pairs in the PDB by Wankowicz and coworkers and Clark and coworkers (Wankowicz *et al.*, 2022 ▸; Clark *et al.*, 2019 ▸), both found that most conformational heterogeneity was present at the side-chain level. In the case that larger, correlated motions are discovered, other available tools will be more suitable. Nonetheless, we integrated some modeling functions from *Coot* to reveal backbone conformational features and overcome the limitations set by simply starting from individual dihedrals. This can be useful for consecutive residues where including subtle backbone flexibility may improve the geometry without generally overparameterizing the model. Our results show that users can have the most confidence in finding rotamers with occupancies of ≥0.25 at resolutions better than 2 Å. Beyond 2 Å resolution, the retrieval of rotamers with occupancies between 0.1 and 0.25 becomes more difficult but may be informative for high-quality or high-flexibility data sets (manuscript in preparation).

One caveat of our approach was found in monitoring clashscores, which is an important metric that is often omitted in validating multi-state models. Models with missing or improperly labeled alternate conformer IDs can inflate clashscores and complicate validation (Richardson *et al.*, 2018 ▸). Although on average *FLEXR* produced models with acceptable clashscores, there were three models with excessively high scores. Manual inspection revealed that these cases were largely driven by *FLEXR* building alternative conformations of bulky side chains in the protein core (Supplementary Fig. S9). Confusingly, while these conformations were justified by *Ringer* plots and represented allowable rotamers, this demonstrated that seemingly genuine *Ringer* peaks can still reflect noise. From a user standpoint this might not be discernable from a true positive peak without placing the alternative conformation in the context of the rest of the protein via building and refinement. A second contribution to high clashscores arises from how *FLEXR* relies on combining peaks in the electron density across dihedrals to construct rotamers, which is inherently ambiguous. This ambiguity can lead to some overbuilding, especially with large, flexible residues such as arginine and lysine, and can create crowded regions with many clashes. Specifically, the *FLEXR* models with the worst clashscores tended to have the most residues with >5 conformers. Hence, we advise the user to inspect these instances and pick relevant conformers carefully. While the automatic removal of seriously clashing side chains is possible, from the perspective of a *Ringer* user we believe that all of these potential rotamers should be built and inspected. This is especially true since we also noticed examples in which weak alternative conformations incorrectly displace water molecules that can be key to understand ligand binding (Darby *et al.*, 2019 ▸; Supplementary Fig. S18). This is a common liability in current refinement programs (Richardson *et al.*, 2018 ▸) and remains so in *FLEXR*. A third contribution to high clashscores can be found in inconsistent alternative conformation labels (also known as ‘altloc ID’). By default, *FLEXR* builds in conformers with the largest electron-density peaks first, so conformers with lower alphabetic IDs generally correspond to the most common conformers. Yet, this approach is ignorant of the space in which the side chain is built so it does not necessarily agree with the conformations of nearby side chains. Although some progress has been made in creating an agreeable network of altloc labels, such as the simulated-annealing approach in *qFit*, it remains an unsolved problem in automated model building.

Investigating model content also revealed distinct modeling patterns between methods. For example, comparing the number of alternative conformations in the deposited and multi-conformer models showed that the deposited models vastly underrepresent side-chain flexibility. Even when alternative conformations are present in the deposited models, they rarely contain more than A and B conformations. While *FLEXR* detected a similar number of flexible residues as the deposited models, it modeled these residues with greater heterogeneity than manually built models since it detects even weakly occupied states. We also observed that *qFit* built nearly identical alternative side-chain conformers about 40% of the time. The large discrepancy in the side-chain content between *qFit* models built with and without backbone sampling suggests that side-chain modeling is biased by other factors, such as, perhaps, satisfying backbone geometry or density. *FLEXR* also produced models with substantially fewer alternative side-chain conformations, <10% on average, than previous *Ringer*-based estimates [∼35% (Fraser *et al.*, 2011 ▸) and ∼18% (Lang *et al.*, 2010 ▸)]. This might be particular to the small test set used here, which also includes lower resolution models than the previous test sets used by Lang and coworkers with resolutions of <1.5 Å. Nonetheless, it is notable that both *qFit* approaches had the opposite behavior and modeled two or three times more than prior estimates. Lastly, about 8% of the alternative conformations built with *FLEXR* had occupancies of zero. Although we observed that generally there is a strong relationship between occupancy and electron-density peak size for serines (Fig. 2 ▸ *c*), this relationship varied greatly when the density peaks are small. We therefore advise against directly inferring occupancy from individual weak *Ringer* peaks, but also discourage disregarding conformers with *prima facie* low occupancy. We only investigated serines for this purpose, since distal atoms in more flexible or branched side chains will confound the relationship between occupancy and electron-density peak height. 50–75% of all low-occupancy conformers (occupancy < 0.25) had acceptable RSCC and *B*_norm_ values (Fig. 2 ▸). Occupancy refinement is an ongoing area of research and several more rigorous methods are available to potentially tease out these subtleties (Pearce, Krojer, Bradley *et al.*, 2017 ▸; Pearce, Krojer & von Delft, 2017 ▸; De Zitter *et al.*, 2022 ▸), which might be key components in biological processes such as ligand binding. Until occupancy refinement improves, the user has the final word in interpreting the conformations in the context of the rest of the structure. The same logic applies to not limiting the number of conformers even though the >5 rotamers per side chain that *FLEXR* sometimes identifies are rarely needed. In the spirit of reducing legwork, deleting unwanted conformers from the model before deposition is easier than including desired conformers manually. When it comes to automated model building, there is no one-fits-all solution. Creating deposition-quality models will require a combination of optimizing *FLEXR* parameters and manually inspecting individual cases: the responsibility is still with the crystallographer (Pozharski *et al.*, 2013 ▸).

Lastly, we also noticed examples in which *FLEXR* detected weak conformations of side chains at the protein–ligand interface that were undetected by other methods. Firstly, it is worrisome that deposited models are missing these features, since binding sites often receive the most attention by crystallographers. Secondly, when using these structures as templates for molecular docking such changes in the protein conformation will produce different small molecules as starting points for drug design (Fischer *et al.*, 2014 ▸). While both approaches are automated, other models did miss these conformations despite clear electron density. In addition to the previously discussed possibilities, another reason for the behavior of *qFit* might be found in the iterative culling process that it uses to remove low-occupancy states (where occupancy < 0.09) between many rounds of refinement. While this has the potential to reduce over-modeling, it might also be liable to removing the same weak conformers that structural biologists are searching for. For instance, the missing alternative Tyr215 conformer in prostaglandin reductase 3 (Figs. 6 ▸ *a*–6 ▸ *c*) could indicate either that the residue contributes less binding energy than anticipated or that the ligand may not be present at full occupancy, with the alternative Tyr215 conformer reflecting the apo state (Pearce, Krojer & von Delft, 2017 ▸). In the process of our analysis, we did not test the effects of varying *qFit* parameters or inspect intermediate *qFit* models that may unearth such features. Like most users would, we used *qFit* with default settings to benchmark the sensitivity and performance of our approach. Likewise, we used *Ringer* parameters that are suggested to maximize sensitivity and to minimize false positives (Lang *et al.*, 2010 ▸). Changing these parameters, especially the electron-density threshold for peak detection and geometric tolerances, will certainly vary the results. While the defaults worked well for most models in the test set, those with high clashscores are examples where these parameters should be optimized through a simple grid search. To facilitate parameter optimization, a script on the *FLEXR* GitHub repository will enable users to perform a grid search.

The overall goal of this project was to produce a tool that automatically incorporates weakly populated, high-energy alternative side-chain conformations from *Ringer* measurements into models. This enables a more thorough examination of local flexibility in the context of the rest of the protein, especially for downstream users of deposited models such as ligand discoverers. *FLEXR* liberates the user from the tedious legwork of detecting conformations and model building. However, the final validation should always be performed by a meticulous crystallographer. *FLEXR* is a fast program that runs well on a personal laptop. It is open-source, freely available and installation is simple (see supporting information). In the context of other model-building tools, *FLEXR* stands as an unassuming tool to explore flexibility in protein structures. Applied to high-resolution structures in the PDB, *FLEXR* can help reveal new information from old data (Touw *et al.*, 2016 ▸; Wankowicz *et al.*, 2022 ▸).

## 5. Data availability

*FLEXR* is open source and publicly available on GitHub at https://github.com/TheFischerLab/FLEXR.

## Supplementary Material

Supplementary Figures, Supplementary Table and Supplementary Methods. DOI: 10.1107/S2059798323002498/qe5002sup1.pdf
