# Supplementary material for: FLEXR: automated multi-conformer model building using electron-density map sampling
Source: Acta Crystallogr D Struct Biol. 2023 Apr 18;79(Pt 5):354–67. doi: 10.1107/S2059798323002498 (PMC10167668; doi:10.1107/S2059798323002498)
Supplement: Supplementary file 1 [file d-79-00354-sup1.pdf]

STRUCTURAL BIOLOGY

Volume 79 (2023)

Supporting information for article:

FLEXR: automated multi-conformer model building using electron-density map sampling

Timothy R Stachowski and Marcus Fischer

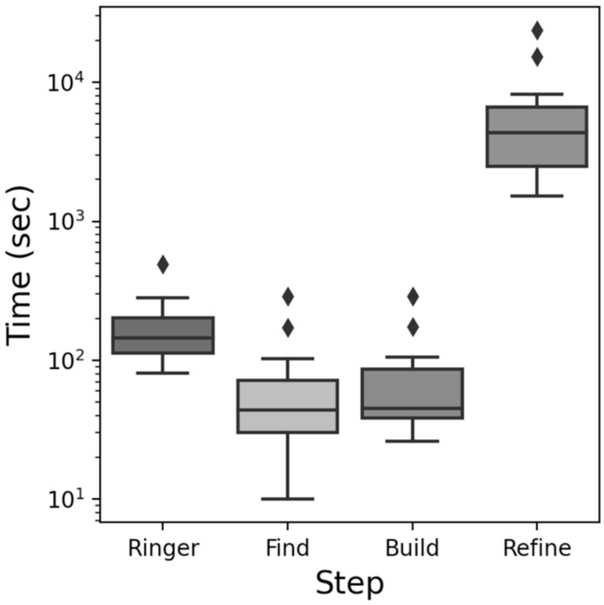

Figure S1 Computation time of the four main *FLEXR* steps.

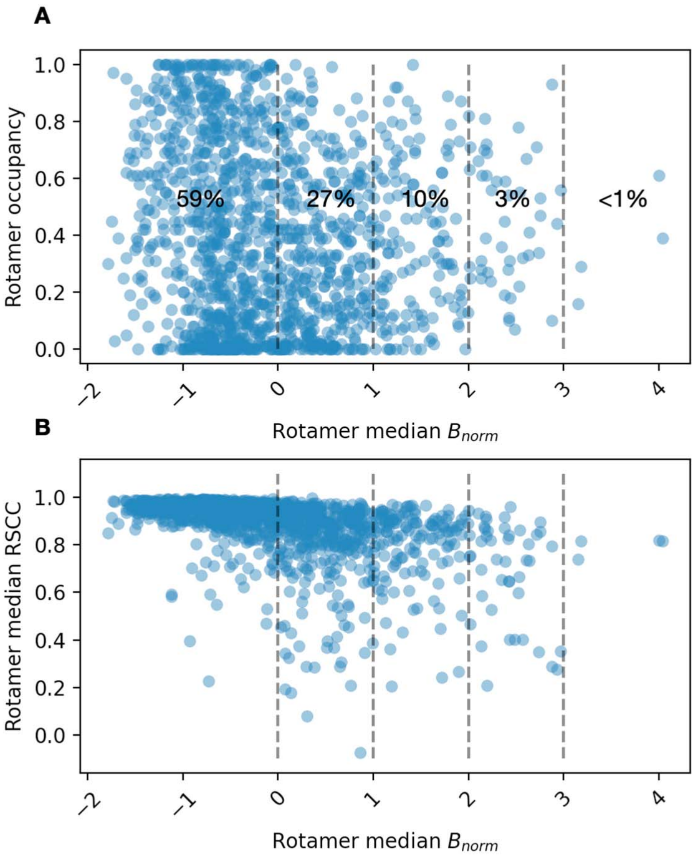

Figure S2 Relationship between B_{norm} and (C) side chain rotamer occupancy or (D) RSCC with dotted lines at certain B_{norm} cutoffs.

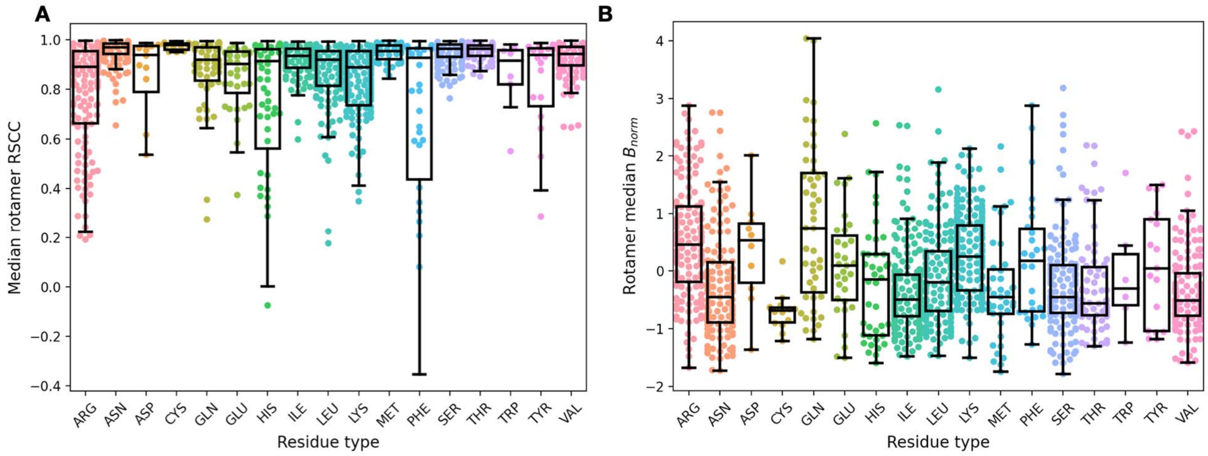

Figure S3 . (A) RSCC and (B) B_{norm} values for rotamers in *FLEXR* models separated by amino acid side chain type.

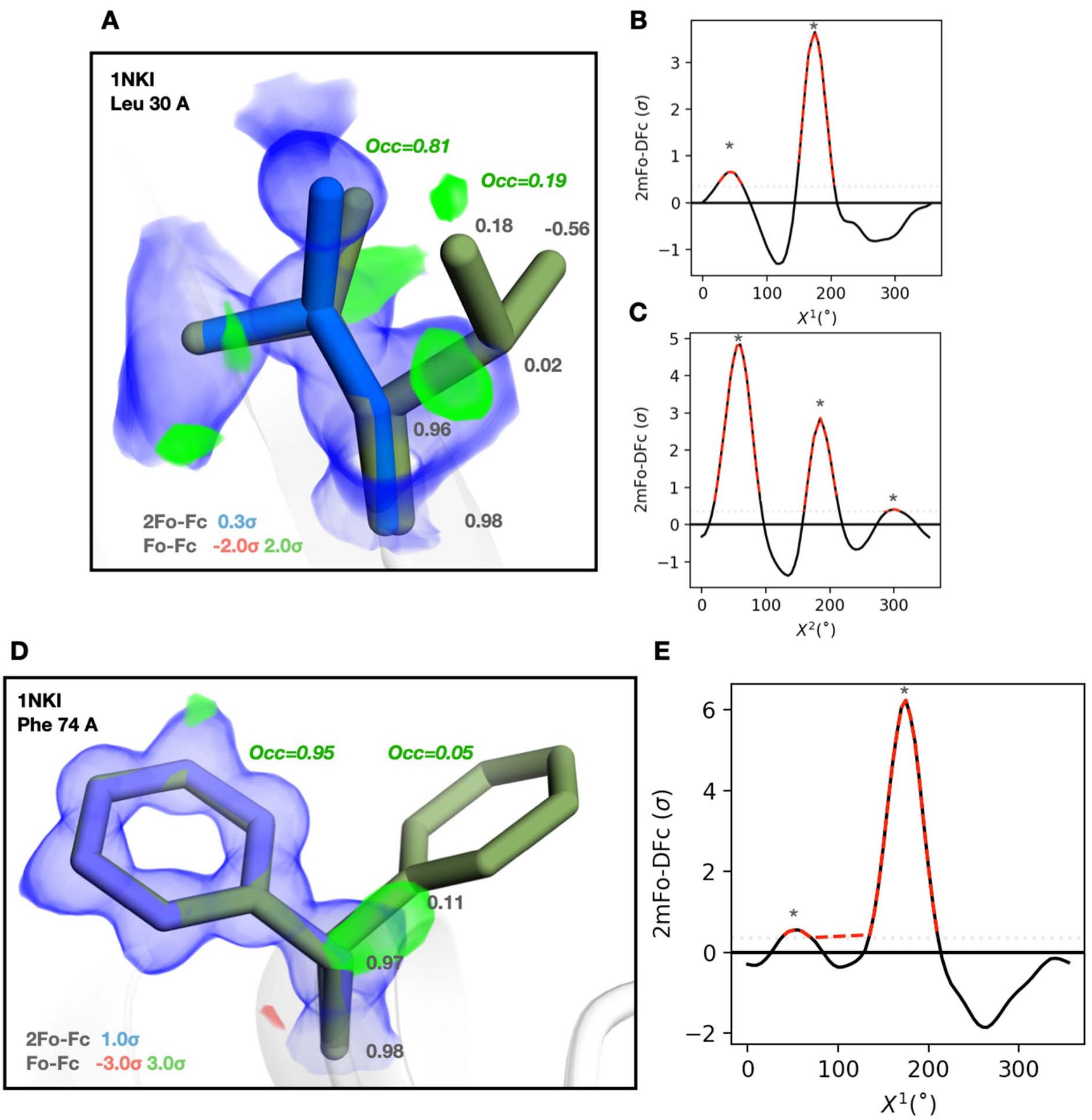

Figure S4 Examples of residues in *FLEXR* models with poor Cγ RSCC values. (A) The Cγ Leu30 from chain A in 1NKI has an RSCC of 0.02 (gray text) but shows clear unmodeled density in both FoFc (green) and 2Fo-Fc (blue) difference maps and (B and C) *Ringer* peaks (measurements in black; measurements above threshold as dotted red; identified peaks marked with gray asterisks). (D) Phe74 from chain A in 1NKI has a weak Cγ RSCC of 0.11 (gray text). A clear Fo-Fc peak (green) and a (E) weak *Ringer* peak both indicate an alternative conformation. Blue sticks represent the deposited model and green sticks represent the *FLEXR* model. Occupancy values are shown in green text. Maps were generated using the deposited structure factors.

Table S1 B-factor refinement approach by each modeling approach

Model (res, Å)	*Deposited*	*FLEXR*	*qFit-nobb*	*qFit*
1w0n, 0.80	ANISO	ANISO	ANISO	na
1nki, 0.95	ANISO	ANISO	ANISO	ANISO
2c6z, 1.20	ANISO	ANISO	ANISO	ANISO
1x9i, 1.16	ANISO	ANISO	ANISO	na
1c9o, 1.17	ANISO	ANISO	ANISO	ANISO
2qd6, 1.28	ANISO	ANISO	ANISO	na
3n6z, 1.30	ANISO	ANISO	ANISO	ANISO
3k0n, 1.39	ANISO	ANISO	ANISO	ANISO
2c0c, 1.45	ANISO	ANISO	ANISO	na
3h0u, 1.50	ISO	ISO	ANISO	na
3el5, 1.60	ISO	ISO	ISO	ISO
3ii7, 1.63	ISO	ISO	ISO	ISO
3ie5, 1.69	ISO	ISO	ISO	ISO
2ozv, 1.70	ISO	ISO	ISO	ISO
3f7w, 1.85	ISO	ISO	na	ISO

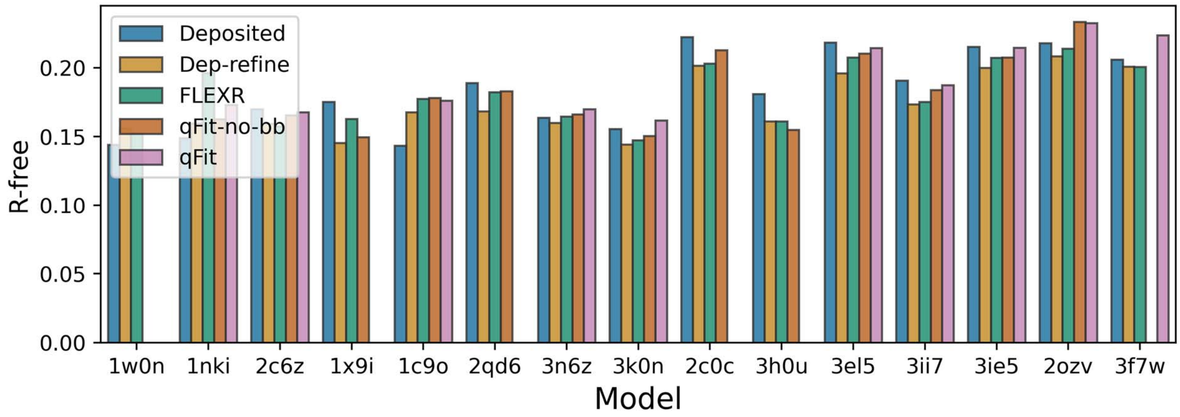

Figure S5 R-free values for individual models. Values are shown for deposited models (blue), re-refined deposited models (light orange), *FLEXR* (green), *qFit* with no backbone (*qFit-no-bb*) sampling (dark orange), and *qFit* with backbone sampling (pink). Missing bars indicate *qFit* runs that failed to converge. Models are organized by resolution from highest (0.8 Å) to lowest resolution (1.8 Å).

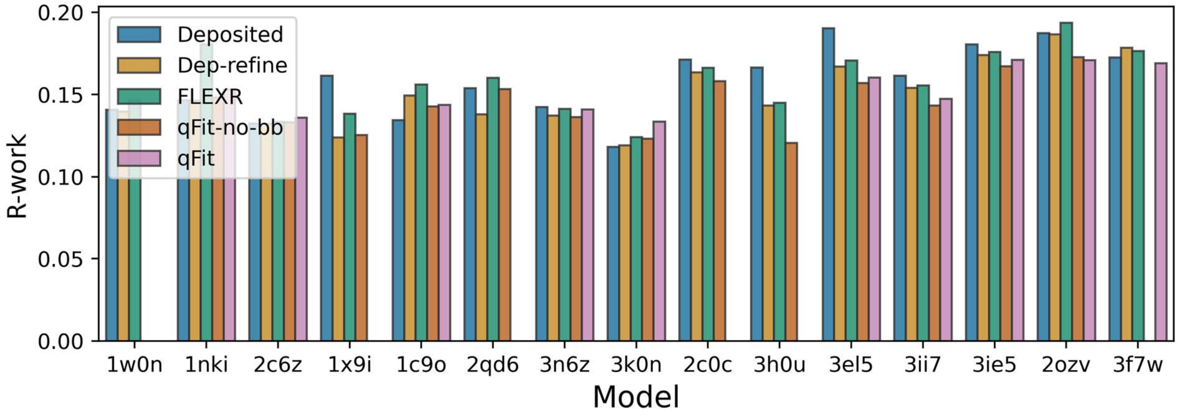

Figure S6 R-work values for individual models.

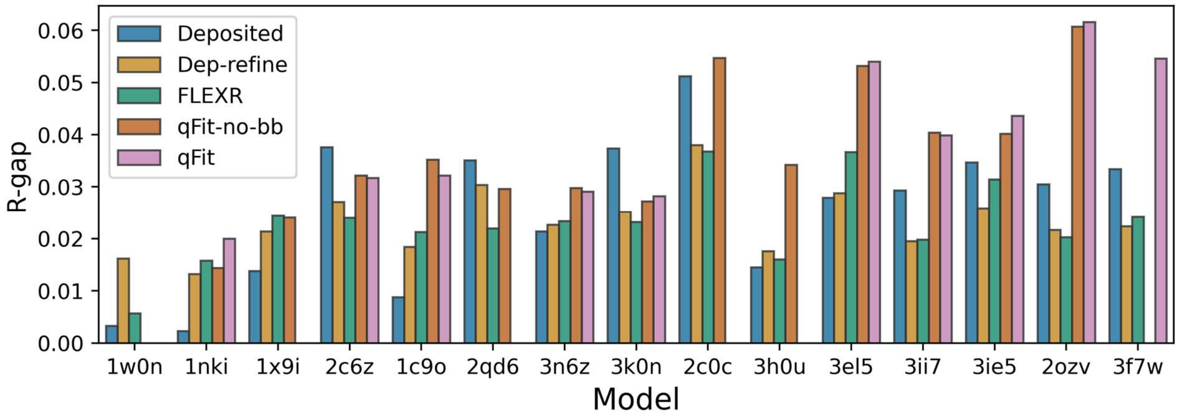

Figure S7 R-gap values for individual models.

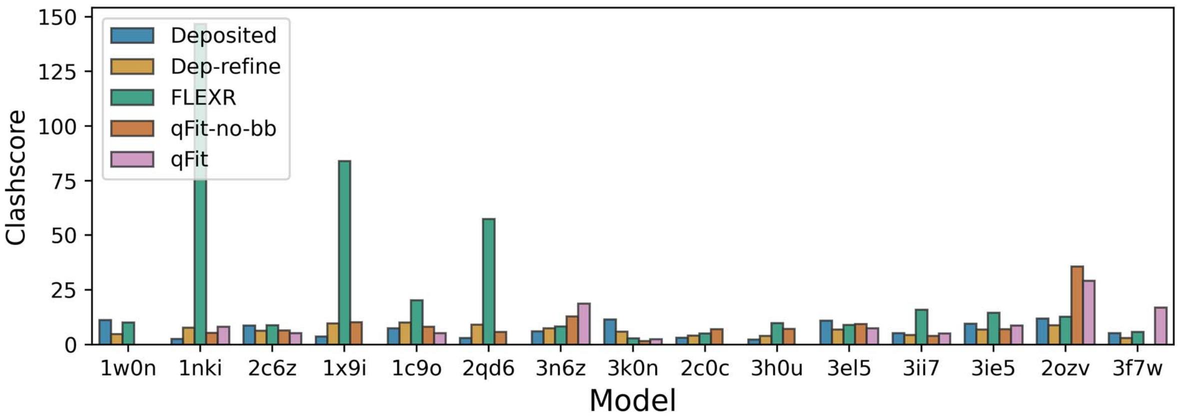

Figure S8 .Clashscores for individual models.

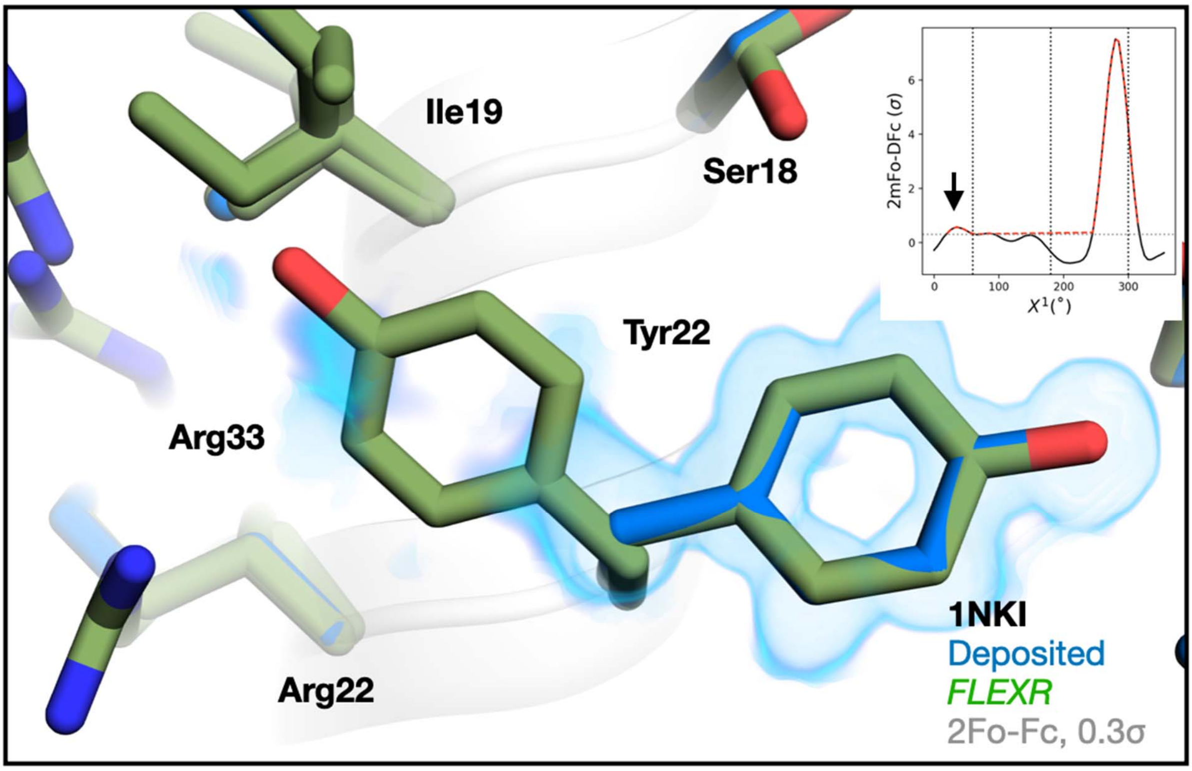

Figure S9 A clash in the interior of 1NKI resulting from the false positive identification of a weak electron density peak. (A) The electron density centered on Tyr22 in chain A in 1NKI. (B) The *Ringer* plot where an arrow indicates a weak alternative conformation. Ideal rotameric angles (60˚, 120˚, and 300˚) are indicated with vertical black dotted lines. The σ threshold is a horizontal gray dotted line and data used for peak detection and integration is the red dashed line. The deposited 2mFo-DFc maps are contoured at 0.3σ (light blue).

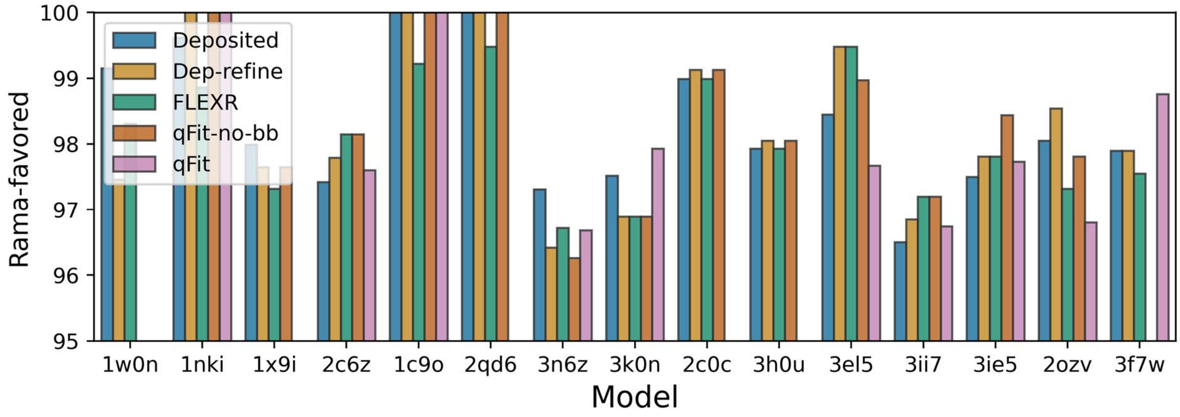

Figure S10 Percent of side chains with favorable Ramachandran geometry for individual models.

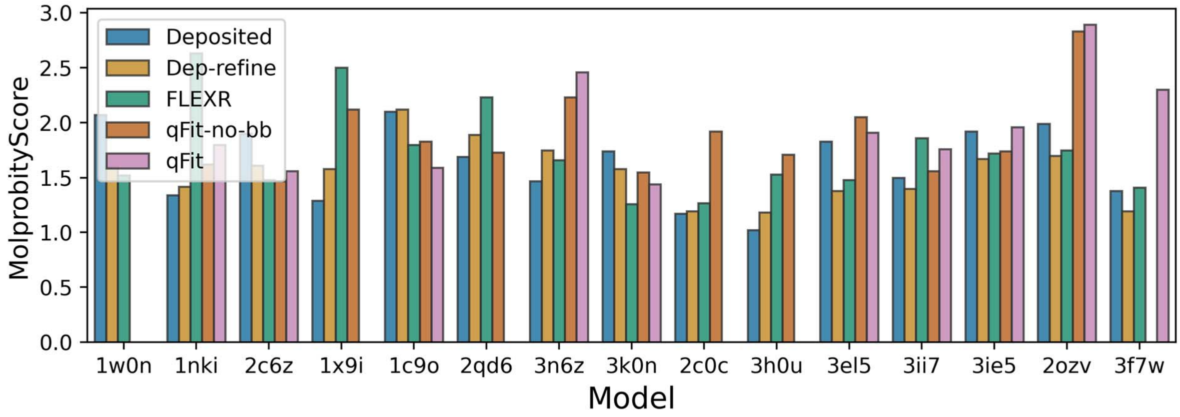

Figure S11 MolProbity scores for individual models.

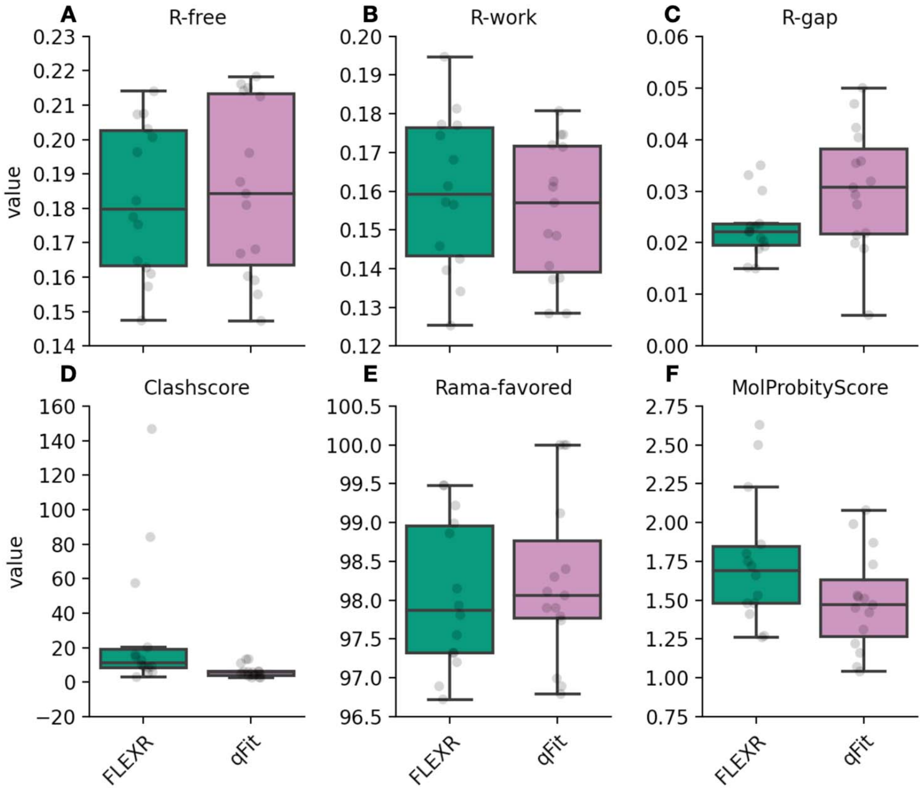

Figure S12 Side-by-side comparison of applying the *FLEXR* (green) and *qFit* (pink; includes low occupancy rotamer culling step) refinement protocols to the final models.

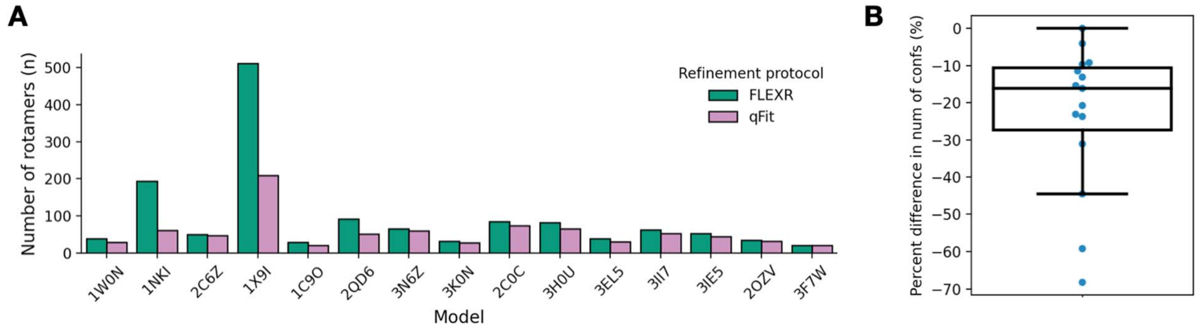

Figure S13 The effect of *FLEXR* vs *qFit* refinement (which includes a low occupancy rotamer culling step) on the number of modeled alternative conformers for (A) each model and (B) precent difference across models.

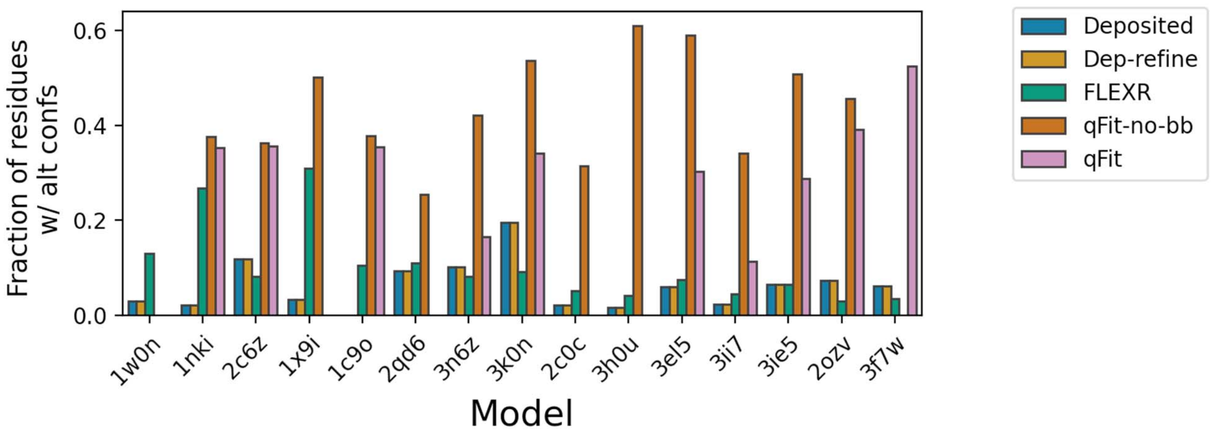

Figure S14 Fraction of residues with alternative conformations for individual models.

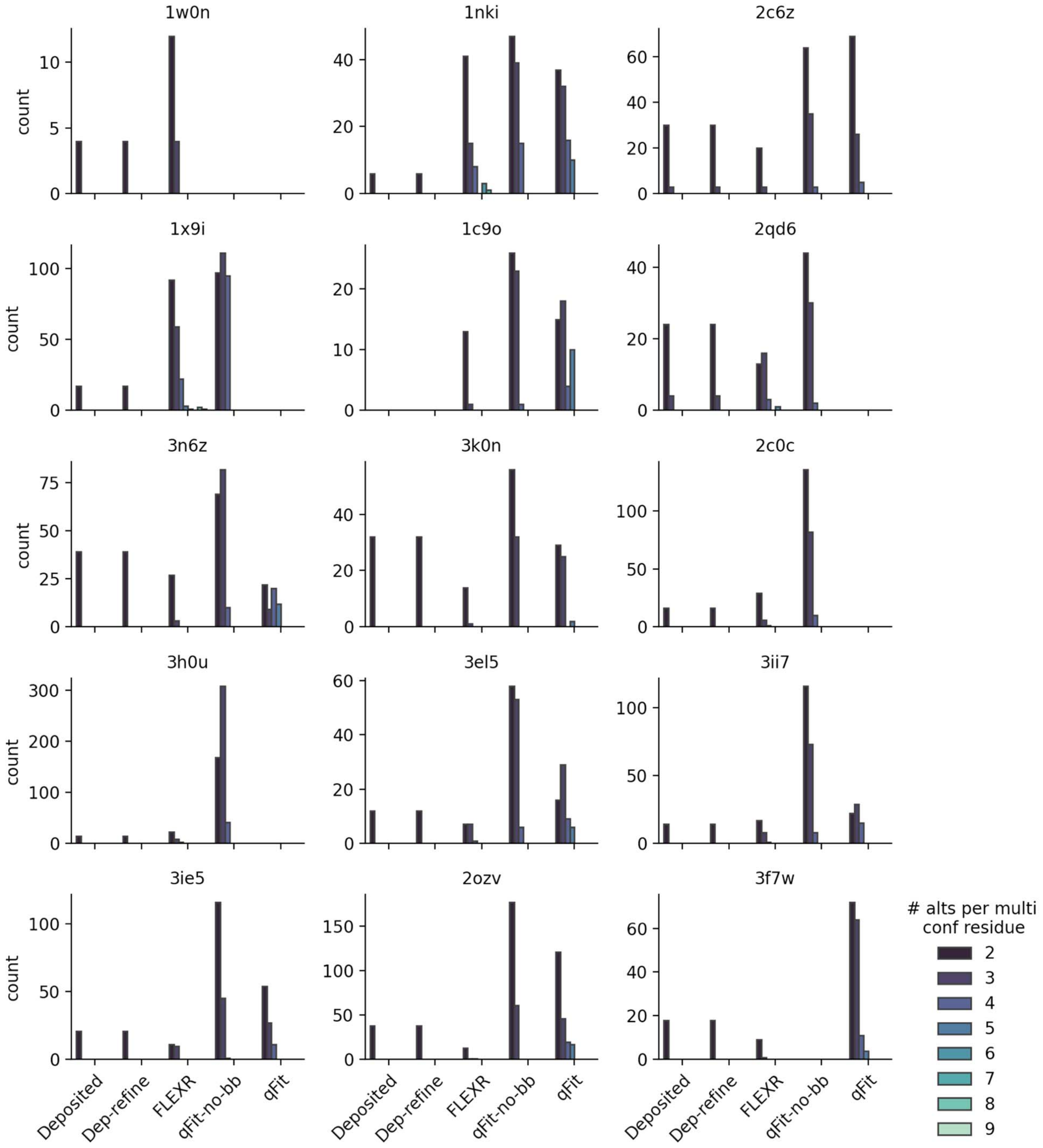

Figure S15 . Distribution of the number of alternative conformations per residue for individual models.

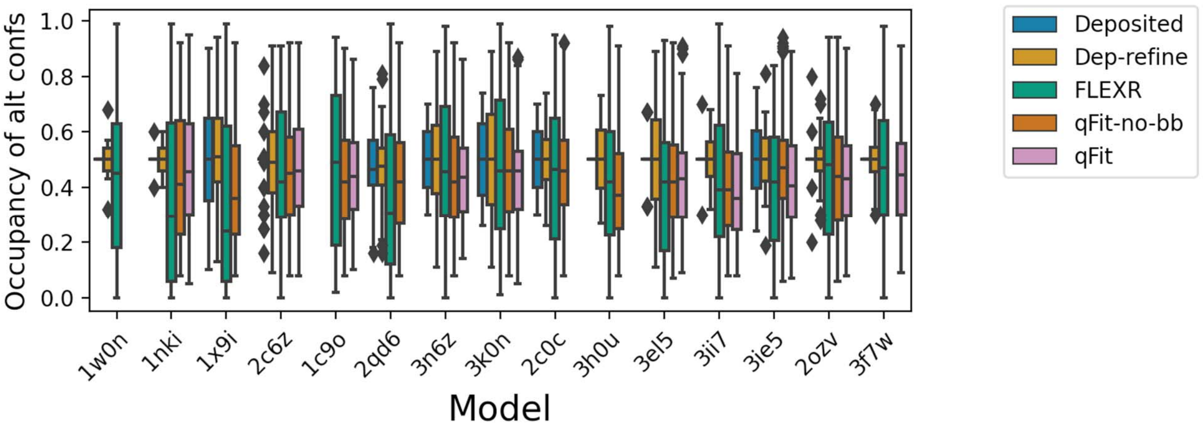

Figure S16 Occupancy per alternative conformation for individual models. Note that the median occupancy of most deposited alternative conformers is 50%.

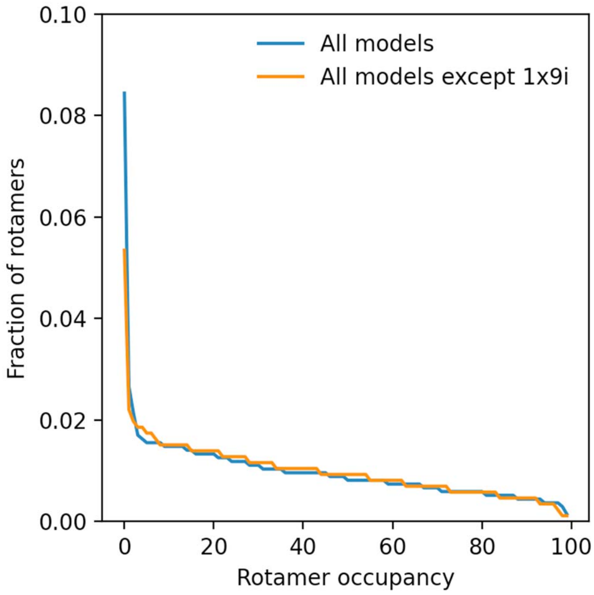

Figure S17 Distribution of rotamer occupancies in *FLEXR* models.

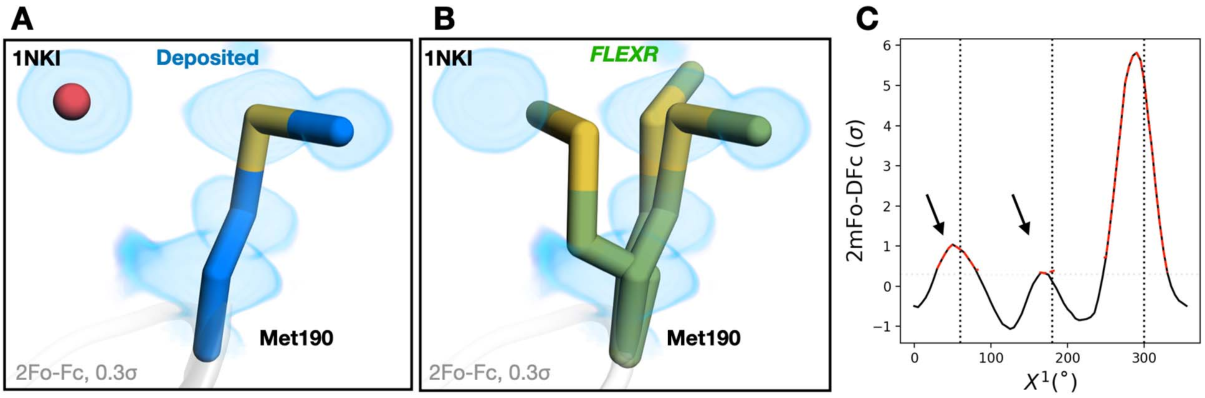

Figure S18 A false positive identification of weak conformers displaces a water molecule in 1X9I. (A) Met190 in chain A in the deposited 1X9I model and (B) in the *FLEXR* model. (C) *FLEXR* identifies three conformations (arrows) for Met190 in the Ringer plot. Ideal rotameric angles (60˚, 120˚, and 300˚) are indicated with vertical black dashed lines. The σ threshold is a horizontal gray dashed line and data used for peak detection and integration is the red dashed line. The deposited 2mFo-DFc maps are contoured at 0.3σ (light blue).

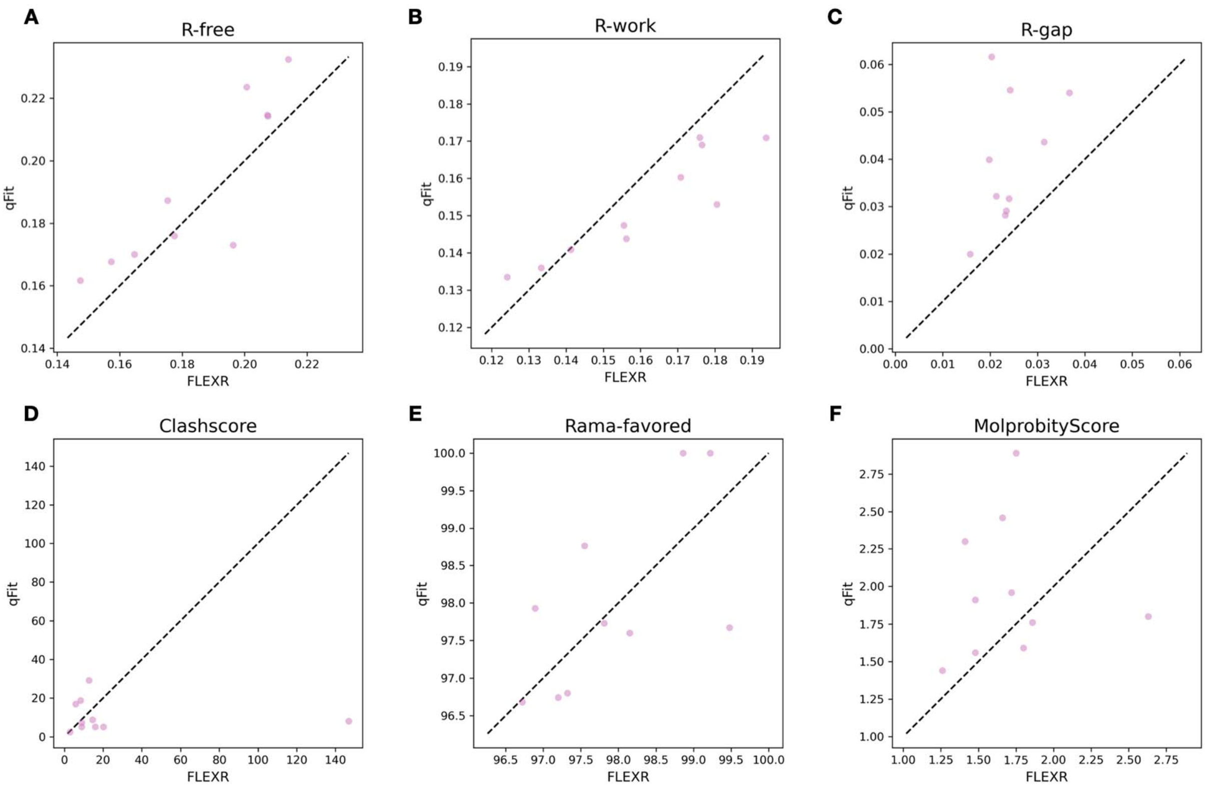

Figure S19 Scatter plots of quality metrics (A-F; cf. SI Figs S5, S6, S7, S8, S10, S11) for individual models (dot) colored by each approach in comparison to *FLEXR*.

S1. Supplementary Methods

FLEXR is a command line tool and is written in Python (v3.9) using Pandas, SciPy, and NumPy packages. Automatic model building is performed using *Coot* (v1.05) (Emsley & Cowtan, 2004). *FLEXR* was tested on MacOS Monterey using an Apple M1 processor. Full documentation can be found at https://github.com/TheFischerLab/FLEXR.

The required Python packages can be installed within an Anaconda environment or separately using pip like:

```
pip3 install numpy
```

Coot should be installed via Homebrew (https://brew.sh) using a formula developed by Yoshitaka Moriwaki:

```
wget https://raw.githubusercontent.com/YoshitakaMo/homebrew-bio/coot/Formula/coot.rb -O coot.rb
```

Coot is then installed with:

```
brew install ./coot.rb
```

Pandas should be installed to the Python version utilized by *Coot* using:

```
/opt/homebrew/bin/python3.10 -m pip install pandas
```

Ringer is available through the MMTBX module of the CCTBX library and comes installed with Phenix (https://phenix-online.org). 2mFo-DFc maps can be calculated from deposited structure factors using:

```
phenix.maps somepdb.pdb somesf.mtz
```

Ringer is then run using:

```
mmtbx.ringer somepdb.pdb  somepdb_map_coeffs.mtz
```

This produces a CSV file, `somepdb_ringer.csv`, which contains the raw electron density measurements that *FLEXR* relies on.

FLEXR can be downloaded by cloning the GitHub repository:

```
git clone https://github.com/TheFischerLab/FLEXR.git
```

The first Python script executed is `flexr_find.py`, which performs three main functions: (i) peak detection from the output electron density measurements from *Ringer*, (ii) assembling possible rotamers, and (iii) checking rotamers against the ideal rotamer library. For (i), several options exist for tweaking the peak finding algorithm like the lower sigma threshold (`-t` flag) and have been previously documented in (Stachowski *et al.*, 2022). For (iii), the main option is geometry tolerance for matching each dihedral (chi) angle detected from *Ringer* plots to ideal geometries. The default value is 30˚ and was defined by (Lang *et al.*, 2010) and can be changed by using the `-g` flag like:

```
python flexr_find.py -f somepdb_ringer.csv -g 40
```

Rotamers scheduled for building are saved to `somepdb_ringer_alts.csv`. Plots showing the electron density and detected peaks and be produced by setting the `-p` flag to `True`.

The second script is `flexr_build.py` , which takes rotamers identified and validated in the previous step and adds to rotamers using *Coot* model building tools and is executed with:

```
/opt/homebrew/Cellar/coot/1.0.05/bin/coot --script flexr_build.py
file.somepdb
```

where ‘somepdb’ is name that matches both the input single conformer model file (`.pdb`) you want to build on and the prefix of the `_ringer_alts.csv` file containing the list of conformers that will be built.

One important setting is whether to add alternative conformations starting at the Cα atom or create an entirely new residue. The default is to create an entirely new residue, but this can be changed using:

```
/opt/homebrew/Cellar/coot/1.0.05/bin/coot --script flexr_build.py \
                                          file.somepdb \
                                          ca_or_all.0
```

Output models are saved to `somepdb_newconfs.pdb` and can be refined using the program of the user's choice. The Phenix PDB editing tool, `phenix.pdbtools`, can be used to filter out rotamers with certain occupancies. For example, to remove rotamers with occupancy=0 use:

```
phenix.pdbtools some_pdb.pdb remove="occupancy=0''
```
